# LEAP-2: An Emerging Endogenous Ghrelin Receptor Antagonist in the Pathophysiology of Obesity

**DOI:** 10.3389/fendo.2021.717544

**Published:** 2021-08-24

**Authors:** Xuehan Lu, Lili Huang, Zhengxiang Huang, Dandan Feng, Richard J. Clark, Chen Chen

**Affiliations:** ^1^School of Biomedical Sciences, University of Queensland, Brisbane, QLD, Australia; ^2^Department of Physiology, Xiangya Medical School, Central South University, Changsha, China

**Keywords:** LEAP-2, ghrelin, GHS-R1a, obesity, growth hormone

## Abstract

Liver-expressed antimicrobial peptide 2 (LEAP-2), originally described as an antimicrobial peptide, has recently been recognized as an endogenous blocker of growth hormone secretagogue receptor 1a (GHS-R1a). GHS-R1a, also known as ghrelin receptor, is a G protein-coupled receptor (GPCR) widely distributed on the hypothalamus and pituitary gland where it exerts its major functions of regulating appetite and growth hormone (GH) secretion. The activity of GHS-R1a is controlled by two counter-regulatory endogenous ligands: Ghrelin (activation) and LEAP-2 (inhibition). Ghrelin activates GHS-R1a on the neuropeptide Y/Agouti-related protein (NPY/AgRP) neurons at the arcuate nucleus (ARC) to promote appetite, and on the pituitary somatotrophs to stimulate GH release. On the flip side, LEAP-2, acts both as an endogenous competitive antagonist of ghrelin and an inverse agonist of constitutive GHS-R1a activity. Such a biological property of LEAP-2 vigorously blocks ghrelin’s effects on food intake and hormonal secretion. In circulation, LEAP-2 displays an inverse pattern as to ghrelin; it increases with food intake and obesity (positive energy balance), whereas decreases upon fasting and weight loss (negative energy balance). Thus, the LEAP-2/ghrelin molar ratio fluctuates in response to energy status and modulation of this ratio conversely influences energy intake. Inhibiting ghrelin’s activity has shown beneficial effects on obesity in preclinical experiments, which sheds light on LEAP-2’s anti-obesity potential. In this review, we will analyze LEAP-2’s effects from a metabolic point of view with a focus on metabolic hormones (e.g., ghrelin, GH, and insulin), and discuss LEAP-2’s potential as a promising therapeutic target for obesity.

## Introduction

In 1996, growth hormone secretagogue receptor 1a (GHS-R1a) was first identified as the receptor of growth hormone secretagogues (GHS) (1). GHSs are small synthetic molecules that amplify pulsatile growth hormone (GH) secretion. The authors defined that GHS-R1a was a G protein-coupled receptor (GPCR) highly expressed in the hypothalamus and anterior pituitary gland (1). Three years later, in 1999, the endogenous ligand of GHS-R1a was isolated from rat stomach by Kojima et al. (2). Kojima and colleagues named this peptide ghrelin after the Proto-Indo-European root “ghre” which stands for “grow” (2). Ghrelin is a 28-residue peptide that is acylated at serine residue at position 3 to generate its active form acyl-ghrelin (2). Kojima verified that ghrelin injection induced GH release from the pituitary gland (2). After that, the orexigenic function of ghrelin was uncovered and identified to act through GHS-R1a on the neuropeptide Y/Agouti-related protein (NPY/AgRP) neurons (3, 4). In a word, ghrelin is an endogenous GHS-R1a agonist that stimulates pulsatile GH secretion and appetite.

Intriguingly, ghrelin had been known as the only endogenous ligand of GHS-R1a for nearly 20 years until 2018 when Ge and colleagues firstly reported liver-expressed antimicrobial peptide 2 (LEAP-2) as an endogenous blocker of GHS-R1a by screening this peptide against a panel of 168 engineered stable GPCR-expressing cell lines (5). Although LEAP-2 was isolated in 2003, it was initially characterized as an antimicrobial peptide expressed in liver (6). After the 2018 report of LEAP-2 action on GHS-R1a, it took an entire year to understand the true mechanism of LEAP-2-GHS-R1a interaction. Initially, a study by Ge et al. identified that LEAP-2 was a non-competitive antagonist of GHS-R1a (5). Subsequent studies revealed that LEAP-2 actually competed for binding sites with ghrelin and also reduced the constitutive activity of GHS-R1a in the absence of ghrelin (7, 8). In 2020, through alanine-scanning mutagenesis, the binding residues and key interactions between LEAP-2 and GHS-R1a were identified, which facilitates the design of novel GHS-R1a antagonists (9).

In terms of the biological actions, in 2019, Mani and colleagues revealed that plasma level of LEAP-2 fluctuated in opposite to that of ghrelin according to metabolic status, and was positively correlated with body mass index (BMI) and many metabolic parameters of obesity (10). Since then, LEAP-2 has been considered as a signal for energy surplus and an efficient regulator of energy balance, therefore has fostered emerging research on LEAP-2’s anti-obesity potent.

However, as a newly recognized GHS-R1a blocker, little is known about its pharmacological actions against obesity. Treating obesity with LEAP-2 may encounter some obstacles, such as its instability and potential side effects on inhibiting GH (5). In this review, we summarize recent findings in the properties of LEAP-2, analyze its association with obesity, and discuss the potential application and limits in anti-obesity. The coverage of interrelationship of ghrelin and GHS-R1a has been limited in this review as they have been thoroughly reviewed earlier (11, 12).

## Biological Maturation and Molecular Structure of LEAP-2

**Figure 1:**
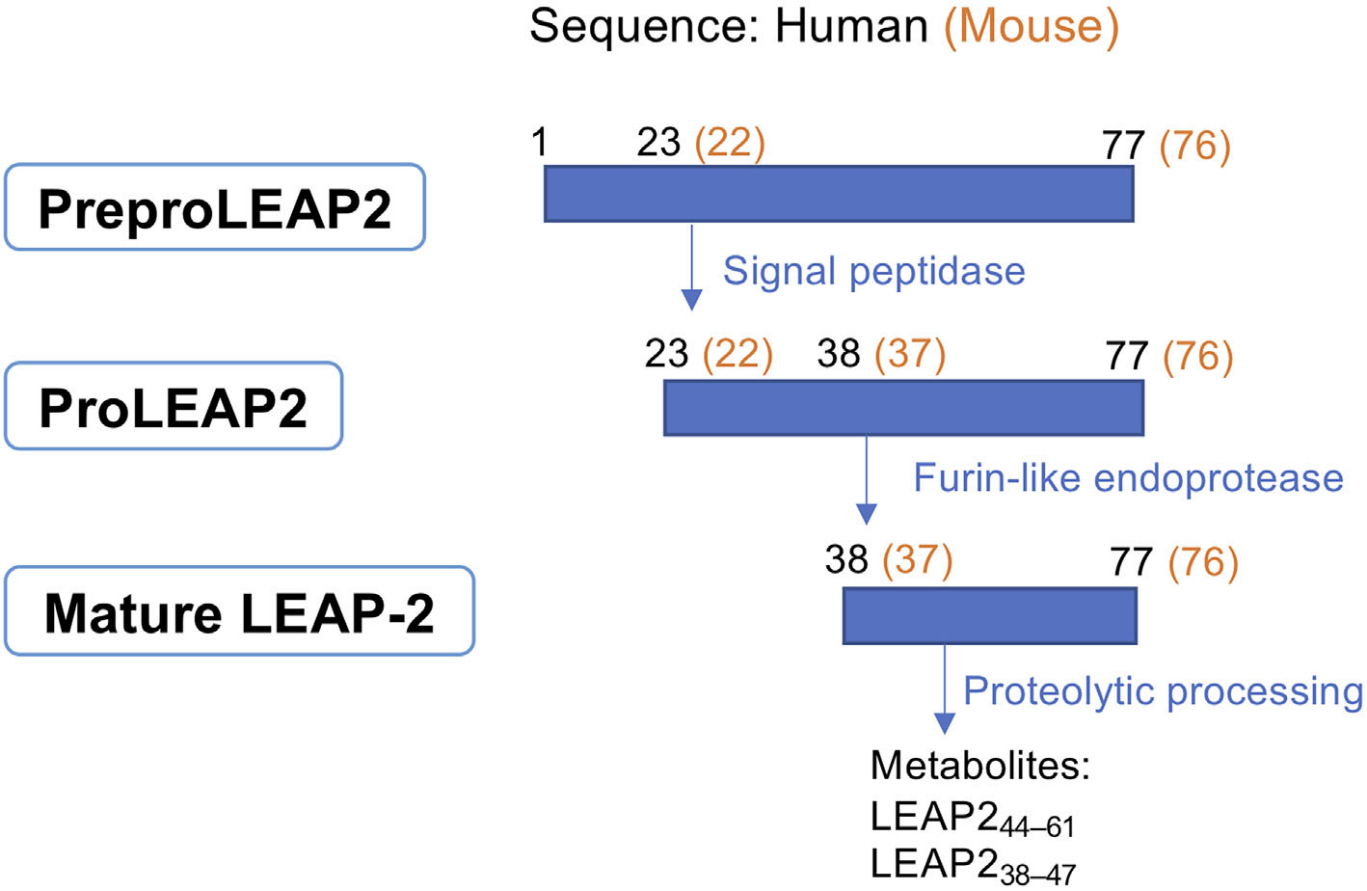
Biological maturation of LEAP-2. PreproLEAP2 is processed into proLEAP2 and then the 40-residue mature LEAP-2 by signal peptidase and furin-like endoprotease. Mature LEAP-2 subsequently degrades into small metabolites through proteolytic processing. The LEAP-2 sequences of human and mouse are labelled in black and orange, respectively.

The biosynthesis of LEAP-2 involves multiple steps. As shown in **Figure 1**, *leap2* mRNA codes for preproLEAP2, a 77- or 76-residue precursor in human or mouse respectively (6). PreproLEAP2 is then processed by a signal peptidase into proLEAP2 (LEAP2_23-77_ of human or LEAP2_22-76_ of mouse). Subsequently, proLEAP2 is cleaved, probably by a furin-like endoprotease, into the 40-residue mature form LEAP2_38-77_ (human) or LEAP2_37-76_ (mouse) (6, 13). The sequence of mature LEAP-2 is highly conserved among mammals, all containing a N-terminal hydrophobic domain, a core region (cationic domain), and a C-terminal domain (10, 13).

The N-terminal of LEAP-2 is essential for both receptor binding and microbial membrane affinity (8, 13). By assessing receptor binding affinity and biological activity of different length of artificial LEAP-2 fragments, M’Kadmi and colleagues demonstrated that the binding determinant of LEAP-2 resided at the 1−8 N-terminal sequence but a longer segment containing at least the 1−12 N-terminal residues had increased binding potency and efficacy (7). Thereafter, a study based on alanine-scanning mutagenesis confirmed that the key residues for LEAP-2 binding were Thr2, Phe4, Trp5, and Arg6 (9). On the contrary, the C-terminal end seems to be irrelevant to both antibacterial activity and GHS-R1a binding, but may play a role in stabilizing LEAP-2 in the plasma through the formation of two disulfide bonds (Cys54-Cys65 and Cys60-Cys70) (6, 7, 13).

LEAP-2 as a peptide is not stable and is rapidly degraded into fragmental peptides by proteolytic processing with a half-life of approximately 15 min (5, 6, 14). An 18-residue peptide, LEAP2_44–61_, is found in the urine of healthy donors as a biological degraded fragment (6). Additionally, a 10-residue fragment, LEAP2_38–47_, is detectable in human plasma at a very low concentration (7.6~11.5 pM) and present in intestinal epithelium of human and mouse. Some fragmental peptides of LEAP-2 are functional, presumably because they retain the N-terminal bioactive sequence, for example, LEAP2_38–47_ has the ability to inhibit GHS-R1a and augment glucose-stimulated insulin secretion (GSIS) from isolated human islets. However, exogenous LEAP2_38–47_ infusion to healthy human subjects did not show any clear physiological effect due to its instability and lower potency (14).

## The Antimicrobial Effect of LEAP-2

LEAP-2 was originally identified as an antimicrobial peptide serving as a part of innate immune system. In response to bacterial infection, LEAP-2 is induced in the liver, small intestine, immune tissues (*e.g.*, bone marrow and tonsil) (15–17), as well as cerebrospinal fluid (CSF) of patients with bacterial meningitis (18). As indicated by its name, LEAP-2 is predominantly expressed in hepatocytes of the liver. The second highest expression level of LEAP-2 is found in the small intestine (highest in jejunum, followed by duodenum and ileum), where LEAP-2 is specifically found in the enterocytes along the epithelial layer (5, 14). In addition, pancreas, pituitary, lung, and kidney also show LEAP-2 expression as detected by qPCR in rats (19). The central nervous system (CNS) also produces LEAP-2, despite relatively lower expression levels, including cerebellum, olfactory bulb, hippocampus, cortex, hypothalamus, midbrain, and medulla oblongata (19).

*In vitro*, LEAP-2 can make holes in the membrane of some Gram-positive bacteria due to its strong basicity and two disulfide bonds (6, 13). In patients with seasonal allergic rhinitis, LEAP-2 expression in the tonsils is reduced, leading to diminished antimicrobial defense. This might explain why allergic rhinitis individuals tend to be more susceptible to upper respiratory tract infection (20). However, the antimicrobial effect of LEAP-2 seems to be predominantly assigned to bacterial infection given that enterocytes expressed less LEAP-2 in response to virus infections by human immunodeficiency virus (HIV) and hepatitis C virus (HCV) (21).

Moreover, LEAP-2 is associated with inflammation. In patients with rheumatoid arthritis, an autoimmune disease, LEAP-2 levels were elevated and positively correlated with C-reactive protein (CRP) and inflammatory cytokines rather than BMI (22). Similarly, CSF LEAP-2 level also had a positive correlation with inflammatory parameters in patients with bacterial meningitis (18). As a result, inflammation may upregulate LEAP-2. Chronic inflammation causes sustained high LEAP-2 level, which subsequently reduces appetite by antagonizing ghrelin action and contributes to cachexia (23).

## LEAP-2 is Both Inverse Agonist and Antagonist of GHS-R1a

Apart from the antibacterial effect, the function of LEAP-2 has been unmasked as both an inverse agonist (reduces constitutive receptor activity) and competitive antagonist (competes for ghrelin binding site) of GHS-R1a (7, 8).

**Figure 2:**
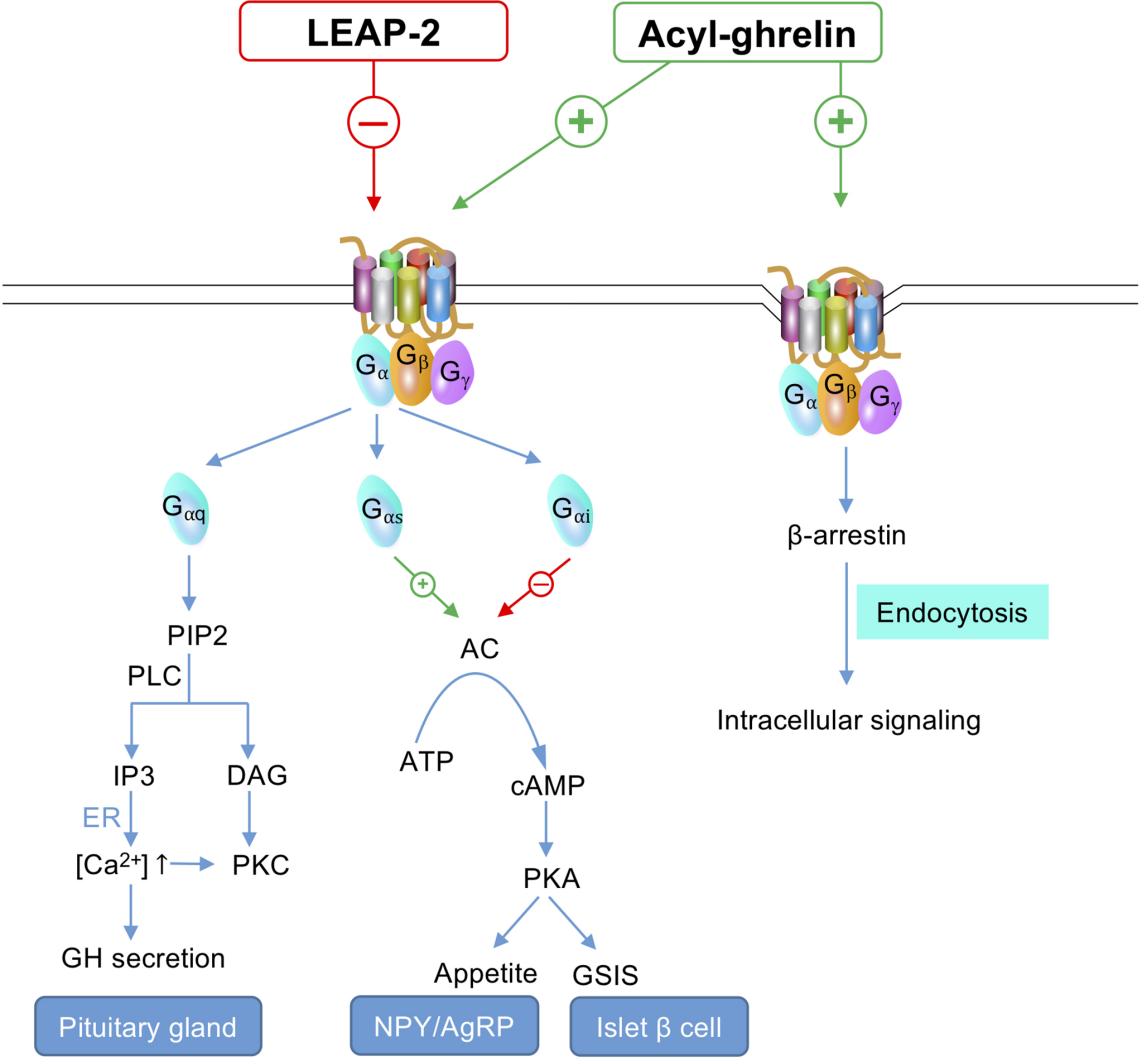
GHS-R1a signaling under LEAP-2 suppression or ghrelin stimulation. G_αq_ – PLC – IP3 cascade on pituitary gland is responsible for GH secretion. G_αs_ – cAMP – PKA pathway on the NPY/AgRP neurons regulates appetite. G_αi_ on islet *β* cells inhibits cAMP – PKA signaling and subsequently prohibits GSIS. Ghrelin initiates GHS-R1a endocytosis through the activity of β-arrestin, which facilitates intracellular signaling, whereas LEAP-2 binding does not lead to receptor internalization. -, inhibit; +, activate; ↑, increase; PIP2, phosphatidylinositol 4,5-bishosphate; PLC, phospholipase C; IP3, inositol (1,4,5) triphosphate; DAG, diacylgycerol; ER, endoplasmic reticulum; [Ca^2+^], Ca^2+^ concentration; GH, growth hormone; PKC, protein kinase C; AC, adenylyl cyclase; ATP, adenosine triphosphate; cAMP, cyclic adenosine monophosphate; PKA, protein kinase A; GSIS, glucose-stimulated insulin secretion; NPY/AgRP, neuropeptide Y/agouti-related protein.

The downstream function of GHS-R1a is mediated through the activation of G protein (G_αq_, G_αs_, G_αi_, or G_α12/13_) and β-arrestin recruitment (**Figure 2**). The orexigenic effect of ghrelin is mainly mediated through G_αs_ - cyclic adenosine monophosphate (cAMP) - protein kinase A (PKA) signaling pathway on the NPY/AgRP neurons, whereas G_αq_ - phospholipase C (PLC) - inositol (1, 4, 5) triphosphate (IP3) cascade on pituitary is the dominant pathway regulating GH release (12, 24).

In the absence of ghrelin, GHS-R1a still maintains a high constitutive activity at about 50% of its maximum capacity (25). The high basal signaling enables GHS-R1a to maintain GH secretion and feeding without ghrelin stimulation (26, 27). Thus, inverse agonists can better block GHS-R1a function compared to competitive antagonists (28). As an inverse agonist of GHS-R1a, LEAP-2 inhibits the constitutive activity of GHS-R1a by stabilizing the receptor to a specific inactive conformation (7). Such an effect of LEAP-2 highlights its premier potential in treating obesity over other synthesized GHS-R1a antagonists. In addition, an electrophysiological study also showed that LEAP-2 could hyperpolarize NPY neurons. Hence, ghrelin-stimulated NPY depolarization was prevented or reversed (10), which also indicated LEAP-2 was a GHS-R1a inverse agonist to modulate appetite centrally.

As an antagonist of ghrelin, LEAP-2 was initially reported as a non-competitive antagonist by Ge et al. (5). This was challenged by Wang’s study which showed that LEAP-2 actually competitively bound to the receptor with ghrelin (8). The difference might be attributed to the different methods used for ligands applications. For example, LEAP-2 was added prior to ghrelin in the experiment of Ge et al. (5). Since LEAP-2 dissociates from the receptor much slower than ghrelin (~15 min vs ~1 min) (8), the preincubation of LEAP-2 occupied the binding sites to prohibit ghrelin binding and masked the competitive binding activity between the ligands. In contrast, Wang et al. unmasked the real binding properties by performing both LEAP-2 pre-treatment and ligands co-treatment, followed by a serial of binding assays and activation assays.

As a competitive antagonist, LEAP-2 shares a common ligand-binding pocket on the receptor with ghrelin and the binding affinities of two ligands are almost equivalent (binding K_i_ = ~1nM) (7, 8). Mutational analysis indicated that Phe279 of GHS-R1a was the common binding site of both LEAP-2 and ghrelin (9). GHS-R1a’s Phe279 and Phe312 residues form hydrophobic interactions with Phe4 of LEAP-2, meanwhile, another hydrophobic interaction and an electrostatic interaction are formed between Phe119 and Asp99 of GHS-R1a and Trp5 and Arg6 of LEAP-2, respectively (9). These critical residues of GHS-R1a (*i.e*., Phe279, Phe312, Phe119, and Asp99) are deeply buried inside the receptor to form a LEAP-2 binding patch which is located on one side of the ligand-binding pocket, whereas on the other side resides the ghrelin binding patch (consisting of Phe279 and Phe286) which links to Phe4 and Leu5 of ghrelin respectively (9).

Unlike ghrelin, which causes GHS-R1a endocytosis upon binding, LEAP-2 binding does not cause receptor internalization (**Figure 2**). This was evidenced using a fluorescent labelled study where LEAP-2 was specifically distributed on the cell membrane of GHSR-expressing cells whereas fluorescent ghrelin was predominantly observed in the cytoplasm (29). It awaits further investigation whether such endocytosis difference for LEAP-2 limits the intracellular signaling of GHS-R1a.

In conclusion, LEAP-2 inhibits the function of GHS-R1a by reducing constitutive activity, displacing ghrelin from binding sites, and minimizing intracellular signaling activated through receptor internalization, and therefore is a specific and potent endogenous GHS-R1a blocker.

## The Counter-Regulatory Effects of LEAP-2 and Ghrelin

**Figure 3:**
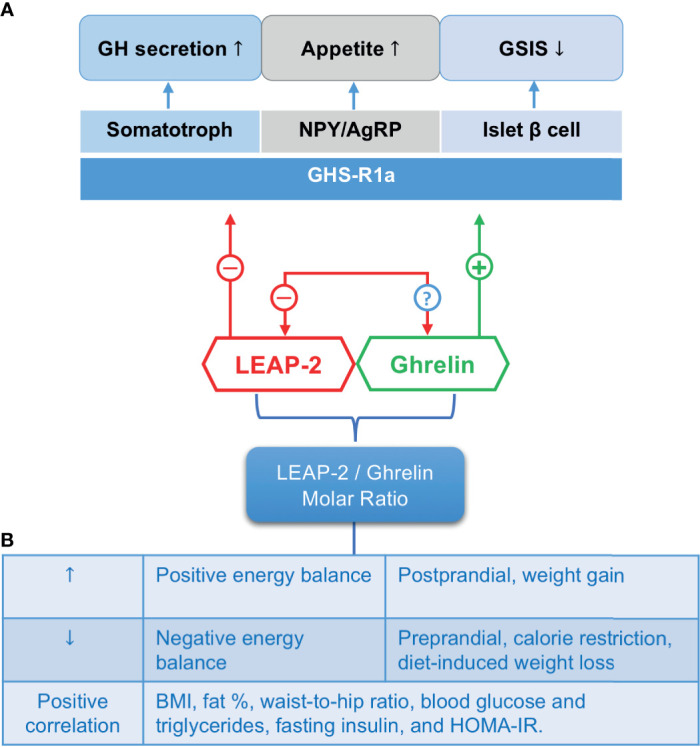
The counter-regulatory effects of LEAP-2 and ghrelin. **(A)** LEAP-2 blocks whereas ghrelin activates the GHS-R1a on target cells (e.g., somatotroph, NPY/AgRP neurons, and islet β cells) to exert biological functions on regulating GH secretion, appetite and GSIS, respectively. Ghrelin inhibits hepatic LEAP-2 mRNA expression, while LEAP-2’s effect on ghrelin expression is still unknown. **(B)** The LEAP-2/ghrelin molar ratio. This ratio changes accordingly to energy status and correlates positively with BMI and some metabolic parameters. ↑, increase; ↓, decrease; -, inhibit; +, activate; ?, unknown; GH, growth hormone; GSIS, glucose-stimulated insulin secretion; NPY/AgRP, neuropeptide Y/agouti-related protein; BMI, body mass index; HOMA-IR, homeostatic model assessment for insulin resistance.

The counter-regulatory effects between LEAP-2 and ghrelin are not only reflected by competing with the receptor, but also by downregulating the counterpart’s expression and function (**Figure 3**). There have been pieces of evidence demonstrating that LEAP-2 may reverse ghrelin’s effects on promoting appetite, releasing GH, maintaining fasting glucose level, suppressing GSIS, as well as regulating body temperature (5, 19).

### Ghrelin Suppresses LEAP-2 Expression in the Liver Through a GHS-R1a - AMPK - Dependent Pathway

Intravenous ghrelin treatment to fasted mice reduced liver-expressed and circulating level of LEAP-2 (19). However, LEAP-2 expression in the ARC or pituitary was not affected by intravenous ghrelin administration (19), suggesting peripheral ghrelin had little influence on central LEAP-2 expression. Nonetheless, the question whether central ghrelin treatment inhibits local LEAP-2 expression awaits further investigation.

In the liver, ghrelin inhibits LEAP-2 expression through a GHS-R1a - adenosine monophosphate-activated protein kinase (AMPK) - dependent pathway (19). To determine whether the inhibition of LEAP-2 expression by ghrelin was dependent on GHS-R1a, Islam and colleagues delivered ghrelin to GHSR-null mice, which failed to suppress LEAP-2 expression in the liver both *in vivo* and *ex vivo* (19). Also, the authors identified that AMPK played an essential role in regulation of LEAP-2 expression in a hepatocarcinoma cell line (Hepa1-6 cell) (19). As one of the key signaling molecules downstream of GHS-R1a cascade, AMPK is a fuel-sensing enzyme that mediates many of ghrelin’s metabolic effects such as promoting catabolism and inhibiting anabolism (11, 30). When activated by GHS-R1a signaling, AMPK is phosphorylated to suppress the activity of sterol regulatory element-binding protein 1 (SREBP1) (31). SREBP1 is a transcription factor regulating lipogenesis, whose binding consensus sequence is also present at the promoter region of LEAP-2 (19). Thus, SREBP1 may be a regulator downstream of AMPK to modulate hepatic LEAP-2 expression, but it remains to be proved by further investigation. In addition, future studies are needed to confirm whether peripheral ghrelin suppresses LEAP-2 expression in other tissues outside of liver (*e.g.*, intestine) through the same pathway.

In terms of LEAP-2’s regulatory action on the circulating level and expression of ghrelin, there is little information to-date. To the best of our knowledge, LEAP-2 overexpression reduced ghrelin levels of the mice under chronic food restriction, but not in the mice under *ad libitum* feeding, indicating LEAP-2 may play a critical role in long-term energy deficiency (5). However, ghrelin expression in the stomach was not detected in this study. Future studies may verify ghrelin expression in response to LEAP-2 treatment.

In summary, although there is evidence for the downregulating effects of the ligands on the counterpart’s expression, the detailed mechanisms remain largely unknown and await further investigation.

### LEAP-2 Suppresses the Orexigenic Effect of Ghrelin

By binding to GHS-R1a on the NPY/AgRP neurons, ghrelin activates downstream G_αs_-cAMP-PKA signaling which initiates Ca^2+^ influx and causes the depolarization of NPY neurons (32). By contrast, LEAP-2 hyperpolarizes NPY neurons to inhibit the activation of the neurons, thereby blunts the orexigenic effect of ghrelin (**Figure 2**) (10).

Since LEAP-2 primarily derives from peripheral organs such as liver and small intestine, it requires to be transported to the NPY/AgRP neurons to exert its central effect on appetite. Although mRNA expression of ghrelin and LEAP-2 are found in the CNS, levels of expression are relatively low (19). How does peripheral LEAP-2 reach central neurons? Whether it is *via* crossing the blood-brain barrier (BBB) or whether it is centrally produced remains unknown.

Although the source of central LEAP-2 remains unclear, effects of LEAP-2 in regulation of food intake centrally have been studied *via* both intracerebroventricular (i.c.v.) delivery and peripheral administration. These reports have been summarized in **Table 1**. Among them, all the experiments involving i.c.v. delivery of LEAP-2 suppressed the orexigenic effect of ghrelin (19, 33). Peripheral LEAP-2 administration blunted ghrelin-induced food intake only when the dose was 3-fold (19), 10-fold (7), and 20-fold (5) higher than peripheral ghrelin dose, and failed to inhibit the orexigenic effect of ghrelin when given equivalent doses (5). Given that the LEAP-2/ghrelin molar ratio is approximately 10~20 during satiated condition (5, 10), one may anticipate that LEAP-2 might only inhibit food intake in a much higher concentration than ghrelin level, perhaps due to the necessity to block the high constitutive activity of GHS-R1a. In addition, the 40-residue LEAP-2 might be harder to cross the BBB compared to the 28-residue ghrelin. As such, it is not surprising that peripheral LEAP-2 treatment failed to block the effect of i.c.v. ghrelin administration, although the dose was 150-fold higher than ghrelin (19). Thus, a high dose of LEAP-2 may be required to reduce food intake during the treatment of obesity, which increases the risk of adverse effects such as suppressing GH. Future studies should carefully monitor the potential side effects when applying LEAP-2 as an anti-obese drug.

**Table 1:** The effect of LEAP-2 on food intake based on experiments carried out from 2018 to 2021.

Treatment			Orexigenic effect		Model	Age (Week)	Ref.
LEAP-2	Interval	Ghrelin	Ghrelin-induced food intake	Other effects on appetite			
**LEAP-2 central administration**							
i.c.v. 10 pmol^#^	0	i.c.v. 60 pmol	↓	NA	C57BL/6 mice	8–12	(29)
i.c.v. 0.6 nmol^##^	NA	NA	NA	Reduced HFD intake in a binge‐like eating protocol	C57BL/6J mice	9‐12	(33)
i.c.v. co-administration of 1 nmol LEAP-2 and 0.1 nmol ghrelin			↓	Did not suppress dark-phase food intake or 14-h fasting-induced food intake	Wistar rats	8	(19)
i.c.v. 1 nmol	30 min	i.p. 5 nmol	↓	NA	Wistar rats	8	(19)
**LEAP-2 peripheral administration**							
s.c. 3 µmol/kg	10 min	s.c. 0.15 µmol/kg	↓	Can inhibit food intake without ghrelin	C57BL/6 mice	12-16	(5)
s.c. 0.15 µmol/kg	10 min	s.c. 0.15 µmol/kg	↔	NA	C57BL/6 mice	12-16	(5)
s.c. 0.6 nmol/g^###^	NA	NA	NA	Did not affect HFD intake in a binge‐like eating protocol	C57BL/6J mice	9‐12	(33)
s.c. 0.6 nmol/g	10 min	s.c. 0.06 nmol/g	↓	NA	C57BL/6J mice	12-16	(7)
i.p. 15 nmol	30 min	i.c.v. 0.1 nmol	↔	NA	Wistar rats	8	(19)
i.p. 15 nmol	30 min	i.p. 5 nmol	↓	NA	Wistar rats	8	(19)
s.c. 3 µmol/kg^####^	NA	NA	NA	↔	NMRI mice	5-6	(14)

i.c.v., intracerebroventricular administration; s.c., subcutaneous injection; i.p., intraperitoneal injection; i.v., intravenous injection; HFD, high fat diet; ↓, decrease; ↔, no change; NA, Not applicable; ^#^Delivering of LEAP-2 or fluorescent-labelled LEAP-2; ^##^Delivering of full-length LEAP-2 and synthesized 12-residue N-terminal LEAP-2 (1−12-NH_2_); ^###^Delivering of LEAP-2 (1−14), a synthesized 14-residue N-terminal LEAP-2 fragment peptide; ^####^Delivering of mature LEAP-2 and LEAP-2 fragments (LEAP2_38-47_, LEAP2_23-47_, and LEAP2_49-77_).

### LEAP-2 Suppresses the GH-Releasing Effect of Ghrelin

In addition to appetite regulation, LEAP-2 also contributes to the modulation of a key anabolic hormone, growth hormone (GH).

Upon binding to GHS-R1a on the pituitary, ghrelin activates the PLC-IP3 pathway to initiate GH secretion (**Figure 2**) (1, 34). GH is secreted in a pulsatile profile characterized by a low level of spontaneous secretion accompanied by periodic pulses (35). The GH profile is displayed by the amplitude and rhythms (*i.e.*, pulse frequency and regularity of GH release) (36). Ghrelin only releases GH stored in pituitary rather than fostering its synthesis (37), thus ghrelin and its synthetic analogs may amplify GH pulses without affecting GH rhythms (38–40). To date, the detection of GH-suppressing effect of LEAP-2 has only been carried out in two sets of experiments (summarized in **Table 2**) (5, 19). LEAP-2 treatments suppressed ghrelin-induced GH secretion in a dose-dependent manner (5, 19), whereas blocking LEAP-2 by monoclonal antibodies caused a rise in GH level (5). It is still unknown whether LEAP-2 would affect the spontaneous secretion and rhythms of GH, which could be directions for further studies.

**Table 2:** The effect of LEAP-2 on GH release based on experiments.

Treatment	GH Detection method	Effect on GH secretion	Model	Ref.
i.p. LEAP-2 (doses range between 0.72~360 nmol/kg), 10 min later, i.v. 6 nmol/kg ghrelin	At 0-, 5-, 10-, 15-, 30-, and 60-min post ghrelin injection	Inhibited ghrelin-induced GH secretion from a dose higher than 7.2 nmol/kg	3–4 months old male C57BL/6 mice	(5)
LEAP-2 overexpression by AAV	1-point GH detection under the condition of a 40% chronic caloric restriction	Both GH and ghrelin levels were lower than control		
i.p. 10 mg/kg BW LEAP-2 monoclonal antibodies following 24h fasting	Sampling at an interval of 20 min for 2h	Increased total and peak GH		
i.v. 15 nmol LEAP-2, 10 min later, i.v. 5 nmol ghrelin	At 0, 15-, and 30-min post ghrelin injection	Inhibited ghrelin-induced GH secretion	8 weeks old male Wistar rats	(19)
1 µM LEAP-2 +/- 0.1 µM ghrelin add to primary pituitary cells	1 point GH detection	Inhibited ghrelin-induced GH secretion		

i.p, intraperitoneal injection; i.v, intravenous injection; AAV, Adeno-associated virus.

Under physiological condition, the metabolic function of GH on glucose metabolism is to increase blood glucose levels by enhancing hepatic gluconeogenesis and glycogenolysis (41). LEAP-2 can blunt the effect of GH on maintaining fasting blood glucose, especially during chronic calorie restriction (5). Mice overexpressed LEAP-2 by adeno-associated virus (AAV) had reduced ghrelin and GH levels. These mice failed to sustain stable blood glucose during the chronic 40% food restriction challenge, thus were found to be moribund and lethargic. GH infusion rescued them by stabilizing their blood glucose levels, which indicated LEAP-2’s regulatory effect on fasting blood glucose was GH-dependent (5). In terms of lipid metabolism, GH promotes lipid catabolism (lipolysis and lipid oxidation) and prevents lipid anabolism (lipogenesis) (41). Although there has been no study investigating the effect of LEAP-2 on GH’s fat-burning action yet, reduced GH level following LEAP-2 treatment may cause reduced fat usage and increased fat storage, hence fosters adiposity. As such, when developing LEAP-2 as an anti-obese drug candidate, researchers should maintain the balance between LEAP-2’s anti-hunger and anti-GH effects.

Following the progress of obesity (pathophysiological condition), the secretion of GH is severely suppressed (42, 43). Previous studies attributed GH suppression to elevated circulating levels of insulin, leptin, and free fatty acids (FFA) (42–46). Given LEAP-2 is dramatically upregulated under obesity (10), it is highly likely to play a prominent role in reducing GH levels. Interestingly, following weight loss, LEAP-2 level drops and GH level rises towards their non-obese physiological levels, which also suggests the recovery of GH secretion without excessive LEAP-2 (10, 47). Nevertheless, the correlations between GH and LEAP-2 await future verification under both physiological and pathophysiological conditions.

### LEAP-2 Antagonizes the Insulinostatic Effect of Ghrelin

In addition to the effect on hypothalamus and pituitary, ghrelin attenuates GSIS by activating GHS-R1a coupled to G_αi2_ on islet β-cells. G_αi2_ decreases cAMP and then activates voltage-gated K_v_ channels (K_v2.1_ subtype), causing repolarization of cell membrane potential after glucose stimulation. Consequently, Ca^2+^ influx is suppressed and GSIS is inhibited (**Figure 2**) (48, 49).

LEAP-2 could abolish ghrelin’s insulinostatic effect when co-treatment of two peptides on islets (7). Another research on the 10-residue N-terminal LEAP-2 fragment (LEAP2_38-47_) also demonstrated vigorous insulinotropic action *in vitro* (14). However, LEAP2_38-47_ neither facilitated insulin secretion nor affected glucose metabolism in healthy human, probably due to its lower potent and instability without the protection of C-terminal disulfide bonds (14). In addition, given that islet ε-cells *per se* can produce ghrelin, exogenous LEAP-2 might not be able to reach a high local concentration around the islet to overwhelm ghrelin’s effect (50, 51). Hence, the insulinotropic effect of full-length LEAP-2, probably with a relatively higher dose, may need to be tested *in vivo* in the future.

## Bio-Manifestation of LEAP-2 Relative to Energy Balance and its Implication to Metabolic Disorders

As endogenous ligands of GHS-R1a, LEAP-2 and ghrelin work coordinately to fine-tune GHS-R1a activity according to changes in energy metabolic states. The regulation of ghrelin during different energy states has been well-illustrated: ghrelin level is elevated during weight loss, calorie restriction, and insulin-induced hypoglycemia, whereas calorie intake and chronic positive energy balance would suppress ghrelin (52). The fluctuation of LEAP-2 is opposite to that of ghrelin: it rises during positive energy balance (postprandial and weight gain) but drops during negative energy balance (fasting and weight loss) (5, 10). As such, an increase in LEAP-2 level often accompanies a decrease in ghrelin in most conditions, and *vice versa*. The LEAP-2/ghrelin molar ratio is hence an indicator of GHS-R1a activity as well as energy states (**Figure 3**).

Here, we summarized LEAP-2 levels in positive or negative energy balance states in **Table 3**. The units present as nanograms per milliliter (ng/mL) in the literature are all converted to nanomolar (nM) according to the molecular weight of LEAP-2 (4.58 kDa) and ghrelin (3.37 kDa for human and 3.31 kDa for rodents). Then, we analyzed the potential links between LEAP-2 and obesity to facilitate future research on the treatment of obesity based on LEAP-2.

**Table 3:** LEAP-2 and ghrelin levels in positive or negative energy balance.

Conditions		LEAP-2 (nM)	Ghrelin (nM)	Molar ratio	Ref.
**Mouse**					
Lean	Fed	2.4 ± 0.4	0.29 ± 0.025	~ 10	(5)
		6.5 ~ 9.8	0.22 ~ 0.52	~ 17	(10)
	Fasting	0.75 ± 0.05	0.7 ± 0.05	~ 1	(5)
	Refeed after fasting	1.4 ± 0.05	0.36 ± 0.025	~ 4	(5)
Obese	Fed	14.0 ~ 17.5	0.37 ~ 0.62	~ 50	(10)
**Human**					
Lean	Fasting	2.6 ~ 3.9	0.07 ~ 0.09	10 ~ 30	(10)
	Fed	2.18 ~ 3.71	0.11 ~ 0.37	10 ~ 20	(10)
Obese	Fasting	3.3 ~ 6.5	0.08 ~ 0.48	25 ~ 150	(10)
	Fed	3.71 ~ 6.98	0.09 ~ 0.10	25 ~ 170	(10)

### Circulating Levels of LEAP-2 Elevate Upon Feeding and Decline During Fasting

LEAP-2 level increases while ghrelin level decreases following meal consumption, shifting the LEAP-2/ghrelin molar ratio towards 10~20 (**Table 3**) (5, 10). Given that the receptor binding affinity of LEAP-2 and ghrelin is equivalent (7), GHS-R1a signaling bias to an “off” status to prevent postprandial overeating. Under fasting state, ghrelin level ascents whereas LEAP-2 level declines, shifting the LEAP-2/ghrelin ratio close to 1. This switches on GHS-R1a activity to facilitate food-seeking behavior and GH release in order to prevent hypoglycemia (5). The decline in LEAP-2 level after fasting is mainly derived from reduced hepatic LEAP-2 expression since fasted rats showed decreased LEAP-2 expression only in the liver but not in the small intestine (19). Interestingly, the molar ratio of LEAP-2/ghrelin does not change much in humans undergoing overnight fasting compared with fed condition (10), probably because overnight fasting is not a harsh enough challenge for humans. Future experiments should investigate the LEAP-2/ghrelin ratio in humans under a longer fasting period. When mice were refed after fasting, the LEAP-2/ghrelin ratio restored to ~ 4 but did not reach the ratio under fed conditions (10~20), enabling the mouse to consume more energy to fight against energy deficiency (5).

In conclusion, the LEAP-2/ghrelin molar ratio is a sensitive indicator of energy status. Modulation of this ratio may affect food consumption, therefore may be a target to treat obesity caused by hyperphagia.

#### Expression Levels of LEAP-2 Are Differentially Regulated by Different Types of Nutrients

Apart from modifying the amount of food consumption, LEAP-2 may be differentially regulated by different types of nutrients. LEAP-2 expression in a human hepatoma cell line was induced and reduced by high-cholesterol sera and polyunsaturated fatty acids (PUFAs) treatments, respectively (53). Cholesterol induces inflammation and is the risk factor for obesity, heart disease, and stroke (54). In contrast, PUFAs are a family of healthy fat with anti-inflammatory effects (55). Given that the antimicrobial peptide LEAP-2 is positively relevant to inflammatory factors (22), cholesterol and PUFAs may affect hepatic LEAP-2 expression by regulating inflammation. Therefore, LEAP-2 level drops or rises to maximize the consumption of essential nutrients or minimize the intake of unhealthy diet, respectively. Future studies may investigate the alterations of LEAP-2 in response to different nutrients *in vivo*.

### LEAP-2 Increases During Obesity

Obese mice and human have a higher LEAP-2 level and a lower ghrelin level than their normal-weight counterparts. Thus, obesity dramatically amplifies the LEAP-2/ghrelin molar ratio. High concentration of circulating LEAP-2 during obesity prevents ghrelin binding to GHS-R1a and contributes to the so called ghrelin resistant phenomenon observed in diet-induced obese (DIO) mice (56).

Adult DIO mice had approximately 2-fold higher LEAP-2 level and about 1.65-fold lower ghrelin level than lean mice control, causing the molar ratio of LEAP-2/ghrelin about 3.3-fold increase in obesity (10). Similar to obesity in mice, human beings with body mass index (BMI) > 40 had significantly higher fasting LEAP-2 level compared with cohorts with BMI < 40. However, LEAP-2 levels showed no significant difference between cohorts with BMI between 25 ~ 40 and BMI < 25 (10). Indeed, LEAP-2 level positively correlated with BMI, and various other metabolic parameters of obesity, including body fat percentage, waist-to-hip ratio, blood glucose and triglycerides, visceral adipose tissue volume, liver lipid content, fasting insulin, and homeostatic model assessment for insulin resistance (HOMA-IR) (10, 57).

Consequently, LEAP-2 may serve as a metabolic signal for energy surplus during chronic and morbid obesity. Hyperphagia obese patients may benefit from switching off GHS-R1a activity by increasing LEAP-2/ghrelin ratio.

### LEAP-2 Levels During Weight Loss

During diet-induced weight loss in mice, there is a reduction in LEAP-2 level and LEAP-2/ghrelin ratio (10). However, controversial results were showed in weight loss following bariatric surgery including Roux-en-Y gastric bypass (RYGB) and vertical sleeve gastrectomy (VSG) surgery (**Table 4**).

**Table 4:** Changes in circulating and expression levels of LEAP-2 and ghrelin post bariatric surgeries.

		Short-term post-surgery	Long-term post-surgery	Ref.
**RYGB**				
LEAP-2	Plasma level	↔	↓	(14)
	Expression	↑ small intestine	NA	(14)
Ghrelin	Plasma level	↓	↑	(58)
LEAP-2/Ghrelin ratio		↑	↓	
**VSG**				
LEAP-2	Plasma level	NA	↓	(10)
	Expression	↑ in stomach ↓ in duodenum	NA	(5)
Ghrelin	Plasma level	NA	↓	(10)
	Expression	↓ in stomach	NA	(5)
LEAP-2/Ghrelin ratio		NA	↔	(10)

RYGB, Roux-en-Y gastric bypass; VSG, vertical sleeve gastrectomy; ↑, increase; ↓, decrease; ↔, no change; NA, Not applicable.

RYGB divides the stomach into a small upper pouch and a larger lower pouch, and then both pouches are connected to rearranged small intestine bypassing the duodenum and a portion of the jejunum. RYGB showed significant effects in reducing body weight and improving metabolic profiles (14). Within 3 months after obese patients receiving RYGB surgery, fasting and postprandial LEAP-2 levels remained unaltered compared to pre-surgery, despite there being an increase in LEAP-2 expression in small intestinal biopsies (14). 3~24 months following RYGB, fasting and postprandial LEAP-2 levels decreased (10), indicating that LEAP-2 might be regulated by a longer term metabolic change following RYGB. Those findings are consistent with the fluctuation of ghrelin levels after RYGB, where fasting total ghrelin dropped in the short term (≤ 3 months) followed by a rise in the long term (> 3 months) after RYGB (58).

Another efficient bariatric surgery, VSG, removes a large portion (~85%) of the stomach along the greater curvature. One month following VSG surgery of DIO mice, LEAP-2 expression was 52-fold amplified in the stomach while declined by 94% in the duodenum, accompanied by decreased ghrelin expression in stomach, as compared with sham surgery controls (5). However, plasma LEAP-2 and ghrelin levels were not tested in this experiment. A clinical study on obese patients undergoing VSG surgery demonstrated a decrease in LEAP-2 level about 12~18 months post-surgery as compared with pre-surgery levels, which might attribute to success in weight loss. As a large portion of the stomach is removed in VSG, it was not surprising to see a reduction in acyl-ghrelin level following VSG, leading to an unaltered LEAP-2/acyl-ghrelin molar ratio (10).

Given that bariatric surgeries rearrange or remove part of the gastrointestinal tract where a large amount of ghrelin and LEAP-2 is produced, the surgery *per se* may disrupt the impact of weight loss on these two peptides. Thus, diet-induced weight loss may be a better model to study the relation between LEAP-2/ghrelin and weight loss.

## Association Between LEAP-2 and Obese-Related Diseases

LEAP-2 is reported to be associated with two obese-related diseases, including non-alcoholic fatty liver disease (NAFLD) and polycystic ovary syndrome (PCOS). Other diseases, including diabetes, cardiovascular diseases, and cancer, are highly related to obesity, therefore are likely to interact with LEAP-2 although has not been investigated yet. This may be a direction for future pathophysiological studies of LEAP-2.

### Non-Alcoholic Fatty Liver Disease

NAFLD is defined as the accumulation of excessive fat (>5%) in the liver of people who drink little or no alcohol. It is a very common disease that relates to numerous metabolic disorders such as insulin resistance, obesity, and type 2 diabetes (59).

In mice with diet-induced NAFLD, hepatic expression and plasma level of LEAP-2 were increased (57). Circulating LEAP-2 is also elevated in patients with NAFLD (57). In consistent with this, an *in vitro* study revealed that LEAP-2 expression was induced by high-cholesterol sera treatment in a human hepatoma cell line (53). To confirm the role LEAP-2 playing in the development of NAFLD, the authors knocked down LEAP-2 using short hairpin RNA (shRNA) in the NAFLD mouse model. The result showed that hepatic steatosis was relieved following the reduction in LEAP-2 level, which resulted from alterations in lipolytic and lipogenic gene expression as well as improved insulin sensitivity (57). The enhanced lipid catabolism may derive from the effect of GH. Although GH level was not tested in this study, it was predicted to be elevated as another experiment blocking LEAP-2 with a monoclonal antibody significantly increased circulating GH level (5). Elevated GH level following LEAP-2 knockdown promotes lipolysis and inhibits lipogenesis, thereby alleviates hepatic steatosis (35). In terms of improved insulin sensitivity following LEAP-2 knockdown, the mechanism is unclear. It is speculated that knockdown of LEAP-2 increases the expression of ghrelin, which subsequently suppresses insulin secretion through GHS-R1a on islets (7, 14). Since hyperinsulinemia occurring in obesity significantly deteriorates insulin resistant states (60), LEAP-2 knockdown may pull down the excessive insulin secretion, thereby improve insulin sensitivity. However, whether upregulation of LEAP-2 affects insulin sensitivity remains to be studied.

### Polycystic Ovary Syndrome

PCOS is a common endocrine disorder in women and is characterized by menstrual dysfunction, ovarian cysts, hyperandrogenism, and obesity. In PCOS patients, obesity rate varies from 50% to 80% (61).

Unlike the reports in other studies, both LEAP-2 and ghrelin levels were reduced in women with PCOS. Surprisingly, LEAP-2 showed an inverse association with BMI, insulin resistance, and free-androgen index of PCOS patients. Furthermore, a lower LEAP-2 level was associated with a higher possibility of developing PCOS (62).

The unusual correlations of LEAP-2 - ghrelin and LEAP-2 - BMI in PCOS women suggest unknown regulators in modulating LEAP-2 levels. Androgen was proven to suppress ghrelin since anti-androgen treatment increased ghrelin levels in obese PCOS patients (63). As hyperandrogenism is the major endocrine disorder of PCOS patients, it is reasonable to suspect that androgen may suppress LEAP-2 independent of ghrelin. A cross-sectional study of 150 children between 3- and 17-year-old revealed that girls showed a higher LEAP-2 level than boys, here LEAP-2 level was independent of BMI, whereas ghrelin did not show this gender dimorphism (64). In addition, LEAP-2 was significantly higher in pubertal than prepubertal girls, while ghrelin level was higher in prepubertal children than pubescents regardless of gender (64). Thus, sex hormones are likely to play a role in regulating LEAP-2. However, another study on 3~12-year-old children failed to confirm this sexual dimorphism in LEAP-2 levels (65). This is probably because girls younger than 12-years-old are most likely in prepubertal age with low LEAP-2 levels, which thereby narrows the gender difference of LEAP-2 levels between boys and girls.

In conclusion, the study of LEAP-2 in PCOS patients and children revealed the potential regulatory effect of sex hormones on LEAP-2. Indeed, children and adult may have distinct LEAP-2 and ghrelin regulatory systems and we discuss below in the following section.

## Controversial Bio-Measures of LEAP-2 in Childhood Obesity

According to available literature data, the regulatory mechanism of LEAP-2 and ghrelin in obesity during childhood may be inconsistent with that in adults. To date, only two detailed studies detected LEAP-2 levels in children (64, 65).

The first cross-sectional study involving a cohort of 82 children between 3 and 12 years old demonstrated that LEAP-2 level was negatively (while positively in adults as discussed above) correlated with BMI z-score (relative weight adjusted for child age and sex), meanwhile, the ratio of acyl-ghrelin/desacyl-ghrelin was upregulated in childhood obesity (65).

The second study with a larger cohort of 150 children between 3~17 years old, however, did not find a significant difference in LEAP-2 level between lean (4.06 ± 1.80 ng/mL) and obese (4.44 ± 1.97 ng/mL) children. Ghrelin level tended to rise in obese children (763.83 ± 33.54 pg/mL in lean VS 875.82 ± 55.46 pg/mL in obesity) without statistical significance, which was somewhat consistent with the result of the first study (65). Indeed, numerous controversial results reported decreased ghrelin levels in obese children (66–69), which might derive from deviation in selected age ranges among different experiments.

To conclude, in childhood obesity, LEAP-2 level is decreased or remains unchanged, which enables children to sustain a higher metabolic rate. LEAP-2 may have diverse roles in the development of obesity at different ages, which should be a direction for further studies.

## Discussion

In this review, we summarized the properties of LEAP-2 as a potent endogenous GHS-R1a blocker and discussed its counter-regulatory effects versus ghrelin on cellular signaling, feeding, and hormonal secretion. We further analyzed the secretion data of LEAP-2 relative to energy balance and examined its implication to metabolic disorders, especially obesity. It is anticipated that increasing the LEAP-2/ghrelin ratio may be a promising target to treat obesity. Suppressing ghrelin’s activity has shown beneficial effects on reducing adiposity in obese rodents in previous studies (70, 71). However, there has been no research investigating the anti-obese effect of LEAP-2 yet. Although LEAP-2 level is upregulated during obesity, this increase seems to be unsatisfactory to suppress appetite and adiposity because it does not stop people from gaining weight. It remains unclear whether an exogenous supplementary of LEAP-2 would meet the satisfaction to reduce appetite and body weight gain. Nevertheless, LEAP-2 may have beneficial effects on preventing weight rebound during weight loss when ghrelin signal is recovered. Moreover, future studies should also investigate whether obese status would be exacerbated without LEAP-2 using a LEAP-2-null mouse model or by neutralizing endogenous LEAP-2.

As natural LEAP-2 is easily and rapidly degraded in the circulation (5), it requires modification for enhanced stability and half-life before being applied as a drug candidate. Researchers may consider masking the potential cleavage sites or replacing the non-binding C-terminal fragment with a structurally more stable segment.

It should not be ignored that LEAP-2 reduced GH secretion (5, 19). In parallel with the fluctuation of LEAP-2, GH secretion is severely suppressed during the progress of obesity but can be recovered after weight loss (42, 44, 47). As a critical metabolic hormone, the reduction in GH level further exacerbates obese states (43, 72). Thus, treating obesity by LEAP-2 may lead to adverse effects related to GH. Pulsatile GH level should be monitored when investigating the anti-obese activity of LEAP-2 in the future. Alternatively, since GHS-R1a couples to different G proteins in NPY/AgRP neurons (G_αs_) and pituitary gland (G_αq_), NPY/AgRP selective GHS-R1a antagonist can be developed to minimize adverse effects on suppressing GH (12, 24). Furthermore, correlation analysis between GH and LEAP-2 levels should be carried out to identify whether GH suppression is dependent on LEAP-2 levels.

In conclusion, LEAP-2 is a potent endogenous antagonist of GHS-R1a which displays an inverse secretory pattern with ghrelin according to energy changes. Due to its effect on suppressing appetite, LEAP-2 has potential to be developed as an anti-obesity drug. To date, the information on LEAP-2 is still somewhat limited. As an “old” peptide with “novel” property, future studies should fully investigate its physiological and pathophysiological roles.

## Author Contributions

CC, LH, and XL contributed to the conception and design. XL searched the literature and wrote the draft of the manuscript. CC, LH, ZH, and RJC revised the manuscript. DF provided suggestions for the manuscript. All authors contributed to the article and approved the submitted version.

## Funding

XL is the recipient of a Research Training Program Scholarship in the University of Queensland. This work is supported by Grants to CC from National Health and Medical Research Council grant (NHMRC, grant number: APP1113494) and University of Queensland.

## Conflict of Interest

The authors declare that the research was conducted in the absence of any commercial or financial relationships that could be construed as a potential conflict of interest.

## Publisher’s Note

All claims expressed in this article are solely those of the authors and do not necessarily represent those of their affiliated organizations, or those of the publisher, the editors and the reviewers. Any product that may be evaluated in this article, or claim that may be made by its manufacturer, is not guaranteed or endorsed by the publisher.
